# Green synthesis of silver nanoparticles using *Eucommia ulmoides* leaf extract for inhibiting stem end bacteria in cut tree peony flowers

**DOI:** 10.3389/fpls.2023.1176359

**Published:** 2023-05-30

**Authors:** Zhanqiang Ma, Kaiyue Zhang, Wei Guo, Weiwei Yu, Junzhe Wang, Juan Li

**Affiliations:** ^1^ College of Agriculture, Henan University of Science and Technology, Luoyang, China; ^2^ School of Environmental Engineering and Chemistry, Luoyang Institute of Science and Technology, Luoyang, China

**Keywords:** bacterial colonization, tree peony, silver nanoparticles, stem blockage, vase life

## Abstract

Tree peony **(** *Paeonia suffruticosa* Andr.) is a popular cut flower among ornamental plants. However, its short vase life severely hinders the production and application of cut tree peony flowers. To extend the postharvest longevity and improve the horticultural value, silver nanoparticles (Ag-NPs) was applied for reducing bacterial proliferation and xylem blockage in cut tree peony flowers *in vitro* and *in vivo*. Ag-NPs was synthesized with the leaf extract of *Eucommia ulmoides* and characterized. The Ag-NPs aqueous solution showed inhibitory activity against bacterial populations isolated from stem ends of cut tree peony ‘Luoyang Hong’ *in vitro*. The minimum inhibitory concentration (MIC) was 10 mg L^−1^. Compared with the control, pretreatments with Ag-NPs aqueous solution at 5 and 10 mg L^−1^ for 24 h increased flower diameter, relative fresh weight (RFW), and water balance of tree peony ‘Luoyang Hong’ flowers. Additionally, malondialdehyde (MDA) and H_2_O_2_ content in pretreated petals were lower than the control during the vase life. The activities of superoxide dismutase (SOD) and catalase (CAT) in pretreated petals were lower than that of the control at the early vase stage and higher at the late vase life. Furthermore, pretreatments with Ag-NPs aqueous solution at 10 mg L^−1^ for 24 h could reduce bacterial proliferation in the xylem vessels on the stem ends by confocal laser scanning microscope (CLSM) and scanning electron microscope (SEM). Overall, pretreatments with green synthesized Ag-NPs aqueous solution effectively reduced bacteria-induced xylem blockage of cut tree peony, resulting in improved water uptake, extended vase life, and enhanced postharvest quality. Therefore, this technique can be used as a promising postharvest technology in the cut flower industry.

## 1 Introduction

Tree peony *(Paeonia suffruticosa* Andr.) is a perennial woody deciduous shrub, which belongs to the family *Paeoniaceae*, section Moutan, and genus *Paeonia* (Liu et al., 2020). There are nine wild species of tree peony, and as many as 2200 cultivars have been cultivated worldwide (Zhou et al., 2014). Tree peony is honored as the “king of flowers” because of its large flowers, beautiful colors and rich fragrance (Zhou et al., 2014). The tree peony has the symbolic significance of peace, prosperity, wealth and auspiciousness and is loved by the world (Zhang et al., 2014; Zhou et al., 2014). Cut tree peony has become a high-grade and high-value cut flower in the world markets. Unfortunately, cut tree peony flowers have a short vase life hindering the development of the cut tree peony industry (Shi et al., 2015; Wu et al., 2017). Therefore, prolonging the vase life of cut tree peony is an excellent practice for promoting the horticultural industry and cut flower exports (Shi et al., 2015).

It is generally believed that the principal reason for reduced vase life is the proliferation of endogenous bacteria in the stem ends of cut flowers (Li et al., 2019; Das et al., 2021; Liu et al., 2021). Proliferating bacteria block the stems and hinder the absorption and transportation of water. The metabolic products of bacteria, such as enzymes and toxic compounds, can accelerate aging in addition to blockage (Xia et al., 2017). Different biocides have been applied to inhibit bacterial growth in vase solutions to extend vase life and improve the horticultural value of cut flowers. Chemical bacteriostatic agents such as chlorine dioxide, silver nitrate, disinfectants, polymers, aluminum sulfate, and sodium benzoate could prevent bacterial reproduction in the vase solution and in xylem vessels of cut flowers (Nguyen and Lim, 2021; Singh et al., 2022). ClO_2_ could inhibit bacterial growth in vase solution and prolong the vase life in the *Gerbera jamesonii* cut flowers study (Lee et al., 2014). Silver thiosulfate, as a holding preservative, could extend the vase life of the cut *Chrysanthemum morifolium* (Sedaghathoor et al., 2020). However, the long-term use of chemical germicides could harm human health and the environment (Rahman et al., 2014; Singh et al., 2022).

Recently, Nanotechnology has been widely used in many fields, including in medicine, the food industry, agriculture and horticulture (Manzoor et al., 2020; Naing and Kim, 2020; Li et al., 2022). Nanomaterials based on metals exhibit cytotoxic activity on microorganisms (Ye et al., 2022; Aliya et al., 2023). Of all the nanoparticles, Ag-NPs have attracted extensive attention in inhibiting various bacteria and retarding the aging of cut flowers because of their strong and broad bacteriostasis (Rai et al., 2009; Manzoor et al., 2020). Many studies have confirmed that treatments with Ag-NPs can alleviate the bacteria-related blockage and extend the vase life, such as *Acacia holosericea* (Liu et al., 2012), *Gladiolus hybridus* (Li et al., 2017), *Gardenia jasminoides* (Lin et al., 2019), *Antirrhinum majus* (Rabiza-Świder et al., 2020b), *Cosmos bipinnatus* (Skutnik et al., 2020), *Gerbera jamesonii* (Liu et al., 2021) and *Dianthus caryophyllus* (El-Attar and Sakr, 2022). Physical and chemical procedures can be applied to prepare Ag-NPs, and chemical procedures are the most frequently employed (Pirtarighat et al., 2019). Still chemical reductants are expensive and can negatively affect the environment. Therefore, it is unsuitable for some research and applications (Zakeri et al., 2021; Pang et al., 2022).

The green synthesis of Ag-NPs from plants is an emerging field of nanobiotechnology. Synthesized Ag-NPs from plants are biocompatible, cost-effective and eco-friendly compared with other methods (Ahmed et al., 2016; Salem and Fouda, 2021; Oves et al., 2022; Nandhini et al., 2023). Different kinds of plant tissue (e.g., petals, leaves, and calluses) extracts are usually used for the green synthesis of Ag-NPs because they have more biological macromoleculars such as polyphenols, proteins, amino acids and enzymes (Rajeshkumar and Bharath, 2017; Jadoun et al., 2021; Khan et al., 2022; Baran et al., 2023). *Crocus sativus* petal extract was used to prepare Ag-NPs that low applied Ag-NPs concentrations could prevent bacterial reproduction in vase solutions and increased approximately two times of vase life of cut *Rosa rugosa* (Solgi, 2014). It has also been reported that Ag-NPs were synthesized using *Artemisia annua* L. callus extracts, and pulse treatments of Ag-NPs at 125, 250 and 500 mg/L for 1 h reduced bacterial reproduction in the stem ends and extended vase life of cut *A. annua* L. (Xia et al., 2017). Additionally, it has been reported that green synthesis of Ag-NPs using *Piper betle* leaf extracts decreased the total no. of the bacterial colony in the vase solution of cut *Gladiolus grandiflorus* flowers. The study revealed that vase solution (4% sucrose and 2 ppm Ag-NPs) reduced the vascular blockage and improved the antioxidative defense of cut *G. grandiflorus* flowers (Maity et al., 2019). However, little research has been conducted on the green synthesis of Ag-NPs to inhibit endophytic bacterial growth in xylem vessels and delay the aging of cut flowers, even less cut tree peony flowers.

*E. ulmoides* is a deciduous tree distributed in China, and is a traditional Chinese medicine. *E. ulmoides* is the only species of *Eucommia* genus belonging to the Eucommiaceae family (Wang et al., 2019). There is evidence that *E. ulmoides* has therapeutic effects on diabetes, hyperglycemia, hypertension, and Parkinson’s disease (Huang et al., 2021). The leaves of the plant contain many kinds of natural active compounds, such as chlorogenic acid, protocatechuic acid, polysaccharides, iridoids, and flavonoids (Bai et al., 2015; Wang et al., 2020a). These bioactive ingredients are capable of reducing Ag^+^ and forming Ag-NPs (Yang et al., 2021; Xi et al., 2022). Moreover, using these bioactive ingredients as reducing agents to prepare Ag-NPs is economical and environmentally friendly. Thus, the synthesis of Ag-NPs using *E. ulmoides* leaf extract is a viable technique, and the synthesized Ag-NPs will provide specific biological effects.

Tree peony ‘Luoyang Hong’ is a typical purplish red-flowered cultivar of Chinese traditional *P. suffruticosa* cultivars. It is particularly appreciated by Chinese and is considered the first choice for cut flower industry because of the better postharvest quality (Jia et al., 2008; Yang et al., 2018). Therefore, green synthesized Ag-NPs using *E. ulmoides* leaf extract was investigated to inhibit endophytic bacterial proliferation on the stem ends and reduce bacteria-induced xylem blockage of cut tree peony ‘Luoyang Hong’. The study analyzed the biological effect of Ag-NPs on cut tree peony ‘Luoyang Hong’ flowers, and explored the influence of pretreated with Ag-NPs on the vase life and postharvest quality of cut tree peony ‘Luoyang Hong’ flowers. The results could provide a novel, practical and environment-friendly method for preserving cut flowers.

## 2 Materials and methods

### 2.1 Materials

The leaves of *E. ulmoides* were collected from the campus of the researcher and transferred to the laboratory. The leaves were dried in an oven (DHG-9203A, Yiheng, Shanghai, China) at 40 °C for 48 h. ‘Luoyang Hong’ flowers were collected from the Luoyang Shenzhou Tree Peony Garden, Henan, China. Cut ‘Luoyang Hong’ with ~30 cm length of stems were collected in the morning at stage I (Stage I, color-exposed phase; Stage II, mouth-open phase; Stage III, early-open phase; Stage IV, half-open phase; Stage V, fully-open phase; Stage VI, early-decay phase; Stage VII, decay phase) (Shi et al., 2015), and placed in a moist foam box, which was brought to Henan Province Key Laboratory of Efficient Cultivation and Comprehensive Utilization of Tree Peony. The vase life and the optimum viewing period of cut tree peony ‘Luoyang Hong’ were defined by Shi et al. (2015).

### 2.2 Green synthesis of Ag-NPs

#### 2.2.1 Preparation of the leaf extract of *E. ulmoides*

The *E. ulmoides* leaf extract was prepared as described by Yang et al. (2021), with some modifications. In brief, the dried leaf was ground into a fine powder using a domestic miller (TS-48, Jingxin, Shanghai, China). Then, the powder (10 g) was extracted with 90 mL of deionized water (DW) at 80 °C for 2 h. Finally, the extract was separated with qualitative filter paper and kept in a brown bottle at 4 °C.

#### 2.2.2 Preparation of Ag-NPs

The synthesis of Ag-NPs was according to Carrillo-López et al. (2014), with some modifications. In brief, silver nitrate (GHTECH, China) aqueous solutions were prepared (10 mmol L^−1^) with DW. The silver nitrate aqueous solution (200 mL) was added to a 250 mL Erlenmeyer flask and heated to 80°C. Then, one milliliter of the *E. ulmoides* leaf extract was added, drop by drop, to the silver nitrate aqueous solution, and under constant stirring at 80 °C for 2 h. After Ag-NPs formed, the color of the solution changed to brown dark (Bahrami-Teimoori et al., 2017). The suspension was centrifuged at 10,000 rpm for 10 min (using Eppendorf centrifuge 5810R, Germany) and washed with DW and 70% ethanol three times after natural cooling. Finally, the sample was freeze-dried in a freeze dryer (SCIENTZ-18ND, Ningbo Xinzhi, China). The obtained powder was Ag-NPs and was characterized for further studies.

#### 2.2.3 Characterization of Ag-NPs

The absorption spectra were measured with a UV-2600i spectrophotometer (UV-vis, Shimadzu, Japan) at 200−800 nm. Fourier Transform Infrared Spectroscopy (FT-IR) spectra were observed to classify the biomolecules in *E. ulmoides* leaf extract and identify the functional group responsible for the Ag-NPs, utilizing IRTracer-100 (Shimadzu, Japan) in the wavenumber region of 4000−400 cm^−1^. The surface morphological features of the Ag-NPs were characterized using SEM (FlexSEM1000, Hitachi, Japan). The crystalline structure of Ag-NPs was studied using a D8 X-ray powder diffractometer (XRD, Bruker, Germany).

### 2.3 Experimental design and treatments

#### 2.3.1 MIC of Ag-NPs *in vitro*

Bacterial community were isolated from the stems of tree peony ‘Luoyang Hong’, and examined as a population in the study. The proximal end stems were removed 0.5 cm first and then excised 1 cm for the subsequent experiment. The surface of 1 cm stems was sterilized in 75% ethanol for 30 s followed by soaking in 6% sodium hypochlorite for 5 min, then washed five times with sterilized water. The 1 -cm stem was further chopped and ground in a sterile mortar. The fragments were removed and resuspended in 1 mL 0.9% (w/v) of normal saline. The suspension was homogenized by vortex mixing for 3 min. An aliquot of 0.1 mL suspensions was transferred to tubes within 10 mL of the Luria-Bertani (LB) liquid medium and different concentrations of Ag-NPs. The cultures were incubated for 24 h at 30 °C and 150 rpm using a bacteriological incubator (BS-S, Guohua, Changzhou, China). Bacterial growth was measured with a spectrophotometer (T6S, Puxi, Beijing, China) by optical density at 600 nm (OD_600_). The MIC was taken as the lowest concentration of Ag-NPs associated with no color change.

#### 2.3.2 Pretreatment with Ag-NPs aqueous solution

The MIC and 1/2 MIC of Ag-NPs were chosen in the experiments to reduce the toxicity to cut flowers. The stems of the cut tree peony ‘Luoyang Hong’ at Stage II were divided into 25 cm in length, and all but the uppermost leaves were removed. A total of 195 samples were placed into 300 mL plastic bottles containing either 200 mL of DW (control) or Ag-NPs aqueous solution for 24 h, one for each bottle. Afterward, the stems treated with Ag-NPs aqueous solution were placed in 300 mL plastic bottles with 200 mL DW, respectively. Each experiment using 65 cut flowers comprised three independent treatments, and each replicate was performed in triplicate. The experiments were conducted in an air-conditioned room at 22 ± 1 °C with a relative humidity of 50–60%. Light intensity was set at about 80 μmol m^−2^ s^−1^ under a 12 h light/dark cycle using scattered light combined with fluorescent lamp lighting.

Ten cut flowers were chosen and measured in each experiment (DW or Ag-NPs aqueous solution) for morphological indicators and water balance at 8:00 a.m. every morning. Five flowers from each group were analyzed for physiological parameters and Ag^+^ content in stems. Additionally, five stems from each group were used every other day to observe bacterial reproduction in xylem vessels with CLSM.

### 2.4 Measurements

#### 2.4.1 Morphological indicators

The flower diameter was measured once each day using a digital caliper. RFW (% of initial) was determined as described by He et al. (2006). Vase life and water balance were analyzed as described by Shi et al. (2015). Water balance (g stem^−1^ d^−1^) is the difference between the water absorbed and lost by each stem every day.

#### 2.4.2 Physiological parameters

The cryopreserved petal tissue (0.1 g) should be placed in an Eppendorf tube (1.5 mL) with 1 mL of 0.05 mol L^−1^ phosphate buffer (pH = 7), followed by four steel balls in the tube and a freezing grinder (Fstgrd-24, Shanghai Jingxin, China) for 1 min. After centrifugation, the supernatant was used to determine SOD activity, CAT activity and H_2_O_2_ content as described by Shi et al. (2015). SOD activity was expressed in units of g^−1^ min^−1^, with one unit inhibiting 50% of the photoreduction of NBT (Hediye Sekmen et al., 2007). One unit of CAT activity was defined as 1 mol H_2_O_2_ L^−1^ min^−1^ over a period of 3 min following a decrease in absorption (Hediye Sekmen et al., 2007). The MDA content of fresh petals was determined as described by Ren et al. (2017).

#### 2.4.3 Ag^+^ content in stems

The bottom, middle, and top stems were cut 2 cm during the vase period, respectively. After 30 min in the oven at 105°C, the samples were dried to a constant weight at 70°C and ground into powder. The dried powders (0.1 g) were acid digested with 5 mL HNO_3_ overnight and then mineralized in a microwave mineralizer. The digests were diluted with ultrapure water to 25 mL and subjected to ICP-OES (Agilent 5110VDV, USA) analysis for Ag^+^ content.

#### 2.4.4 CLSM observation

The living bacteria in the xylem vessels were observed using CLSM. Proximal end segments (~0.5 cm in length) were excised from the cut tree peony ‘Luoyang Hong’ stems every other day. Cross and longitudinal sections of stems were obtained with a Leica CM 1950 cryostat at a cutting interval of 60 µm thickness. The sections were collected and laid on slides. Each slide was stained with about 50 µL of 20 µmol L^−1^ SYTO9 green fluorescent dyes (Kaixin Biotechnology Co., Ltd., Xian, China) for 20 min in the dark and observed at all times to prevent drying. Subsequently, the samples were analyzed by CLSM (FV3000, Olympus, Tokyo, Japan) at 480 nm excitation and 530 nm emission.

#### 2.4.5 SEM observation

The presence of bacteria in the xylem vessel at the stems end of the cut tree peony ‘Luoyang Hong’ flowers was observed by SEM (JSM-IT200(LA), JEOL, Tokyo, Japan) as described by Li et al. (2017) and Thakur et al. (2022), with some modifications. In brief, Cross-sections of stems (~2 mm length) were excised from the base of each stem using fresh surgical blades every other day. The stem segments were immediately immersed in 0.1 M phosphate buffer (pH 7.2, PBS), and then transferred to 2.5% glutaraldehyde for fixation 6 h at room temperature. After fixation, the stem segments were washed three times with PBS followed by dehydration using graded ethanol series (30%, 50%, 70%, 90% and 100%) of 15 min for each ethanol concentration. The stem segments were eventually dried in a critical point dryer (K850, Quorum Technologies, UK) with liquid CO_2_. Then, the stem segments were mounted with double-sided carbon tape on SEM stub and coated with platinum using a sputter-coater (JFC-1600, JEOL Japan) for 30 s. The images of sample surfaces were observed at 20 kV accelerating voltage for capturing the digital micrographs.

### 2.5 Statistical analysis

An analysis of variance (ANOVA) was conducted to compare treatments using IBM SPSS Statistics 26.0. The least significant difference (LSD) test was used to compare treatment means at *p* ≤ 0.05, and the data were presented as means ± standard errors. Diagrams were constructed using Origin 2018.

## 3 Results

### 3.1 Synthesis of Ag-NPs

**Figure 1:**
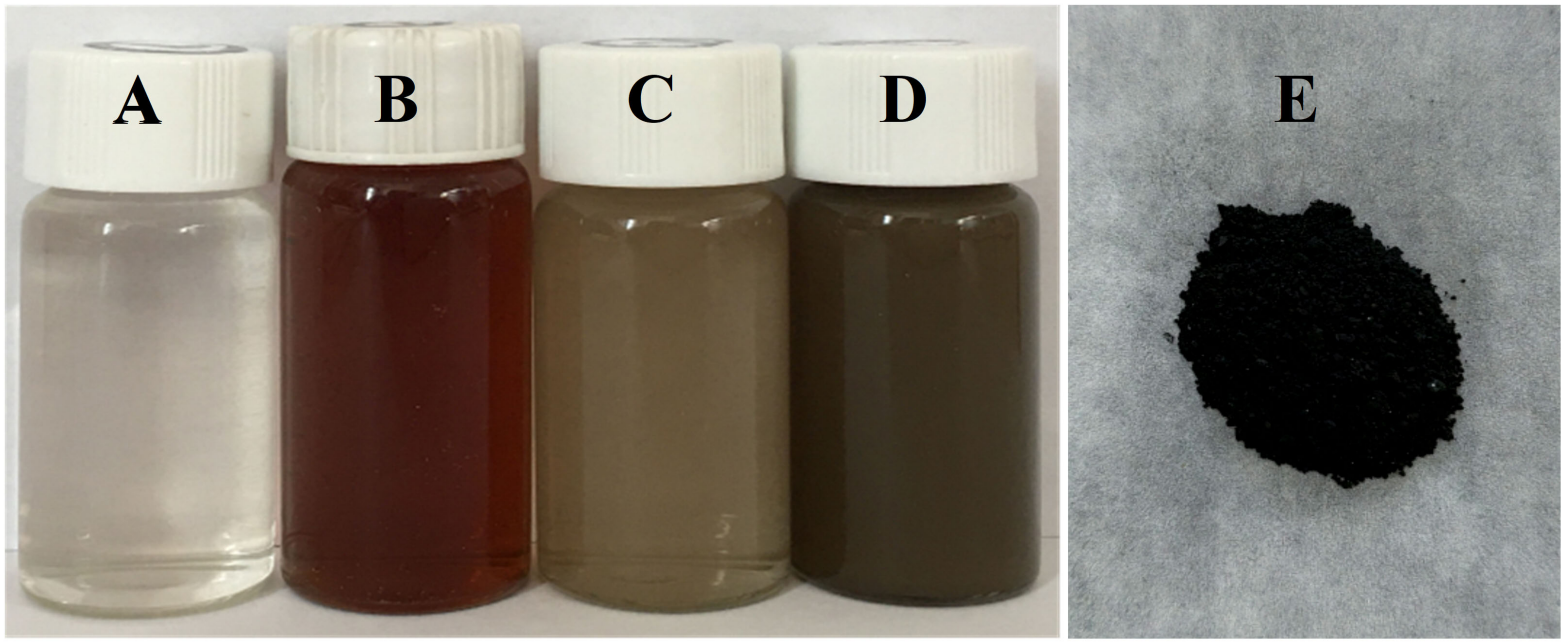
Green synthesis of Ag-NPs. **(A)** solution of silver nitrate, **(B)** *E. ulmoides* leaf extract, **(C)** solution at 10 min of reaction, **(D)** solution at 2 h of reaction, and **(E)** Ag-NPs powder.

**Figure 2:**
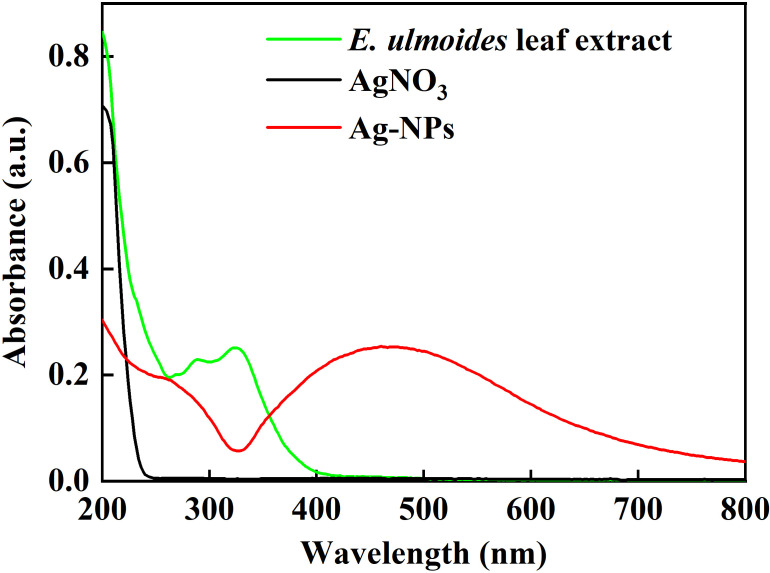
UV-Vis spectra of Ag-NPs.

A color change was observed during the green synthesis of Ag-NPs (**Figure 1**). Silver nitrate solution (**Figure 1A**) and the leaf extract of *E. ulmoides* (**Figure 1B**) were colorless and red brown, respectively. When reacted from 10 min to 2 h, the solution color changed from gray to brown black (**Figures 1C, D**). The maximum absorption peak was at 450 nm (**Figure 2**), confirming the formation of Ag-NPs (Pirtarighat et al., 2019). In contrast, no absorption peak was found for silver nitrate and the leaf extract of *E. ulmoides* at 450 nm. Finally, the solution was freeze-dried, and the obtained black powder was Ag-NPs (**Figure 1E**).

### 3.2 Characterization of Ag-NPs

**Figure 3:**
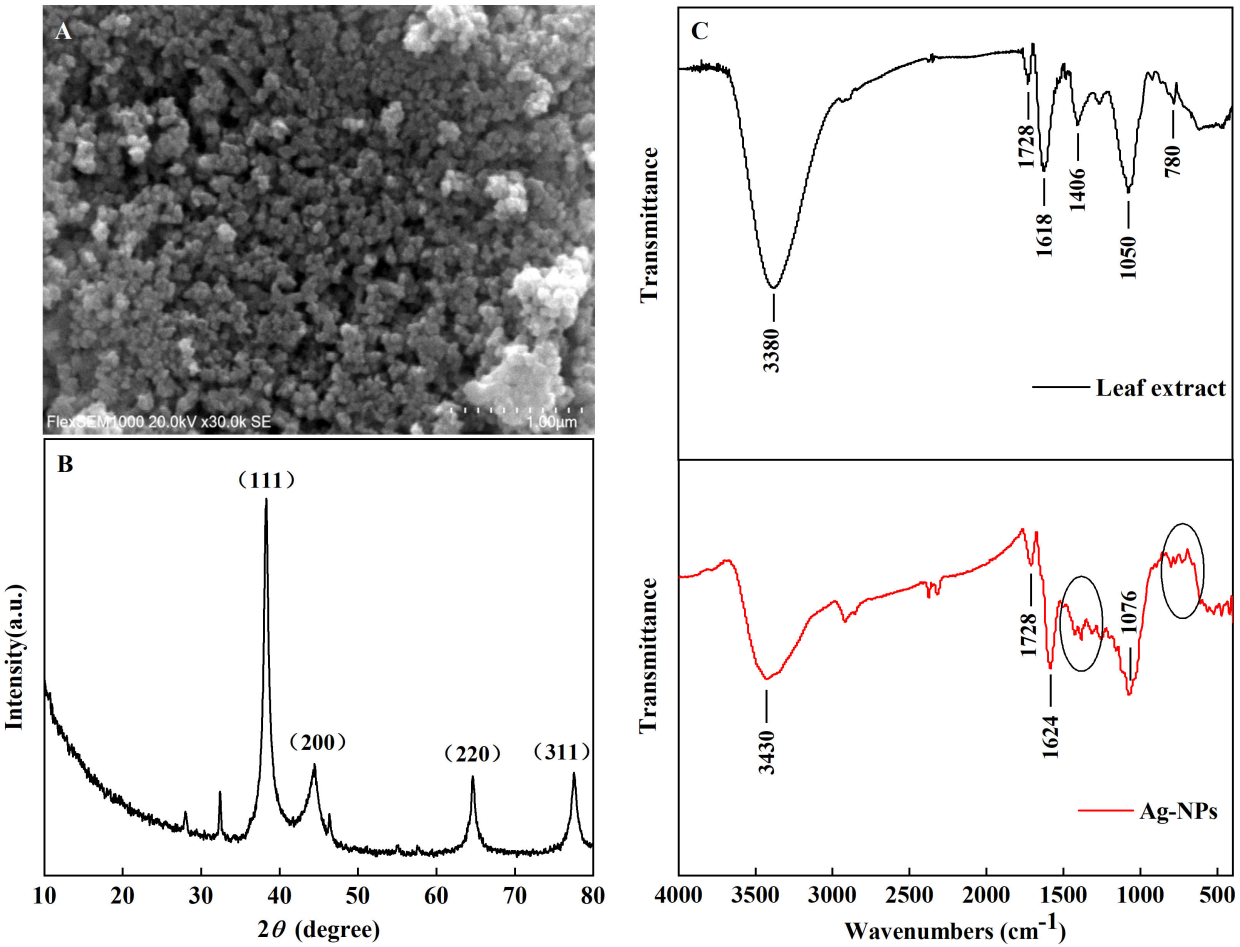
Characterization of Ag-NPs. **(A)** SEM image, **(B)** XRD spectra, and **(C)** FT-IR spectra.

The morphology of the Ag-NPs was observed using SEM. The particles of Ag-NPs were spherical and well distributed, and the particle size was approximately 30~60 nm (**Figure 3A**).

The XRD patterns of the Ag-NPs were carried out (**Figure 3B**). The peaks at 2*θ* of 38.3°, 44.6°, 64.8° and 77.5° are associated with the (111), (200), (220), and (311) planes of Ag, respectively. The peaks were consistent with face-centered cubic crystalline Ag (JCPDS 87−0720) (B Aziz et al., 2019). Other peaks at 2*θ* of 27.9°, 32.4° and 46.3° may result from organic compounds in the leaf extract of *E. ulmoides*, which were used in the synthetic process of Ag-NPs. Previous studies have shown similar results (Vo et al., 2020).

The functional groups were investigated using FT-IR spectra in the *E. ulmoides* leaf extract (**Figure 3C**). The spectra of Ag-NPs showed the disappearance of peaks at 1406 cm^−1^ (C–H, vibration, alcohols) and 780 cm^−1^ (C–H, out of plane vibrations, aromatic) compared with the spectra of the leaf extract of *E. ulmoides*. There might be the reaction of biomacromolecules in the leaf extract of *E. ulmoides* containing carboxylic acids and aldehyde groups with Ag^+^, leading to the formation and stabilization of Ag-NPs. In addition, the spectra of Ag-NPs showed absorbance peaks at 3,430 cm^−1^ (N–H stretch, primary amine of the protein), 1,728 cm^−1^ (C=O, stretch, amide), 1,624 cm^−1^ (N–H, stretch, amines) and 1,076 cm^−1^ (C–O, stretch, ether aromatic). The results illustrated that the functional groups from the extracts were capped with Ag-NPs. It also indicated biomolecules in the leaf extract of *E. ulmoides* such as proteins, aldehydes, polyphenols, amino acids, and other biomolecules, contributed to the reduction of Ag^+^ to Ag-NPs.

### 3.3 MIC of Ag-NPs *in vitro*

**Figure 4:**
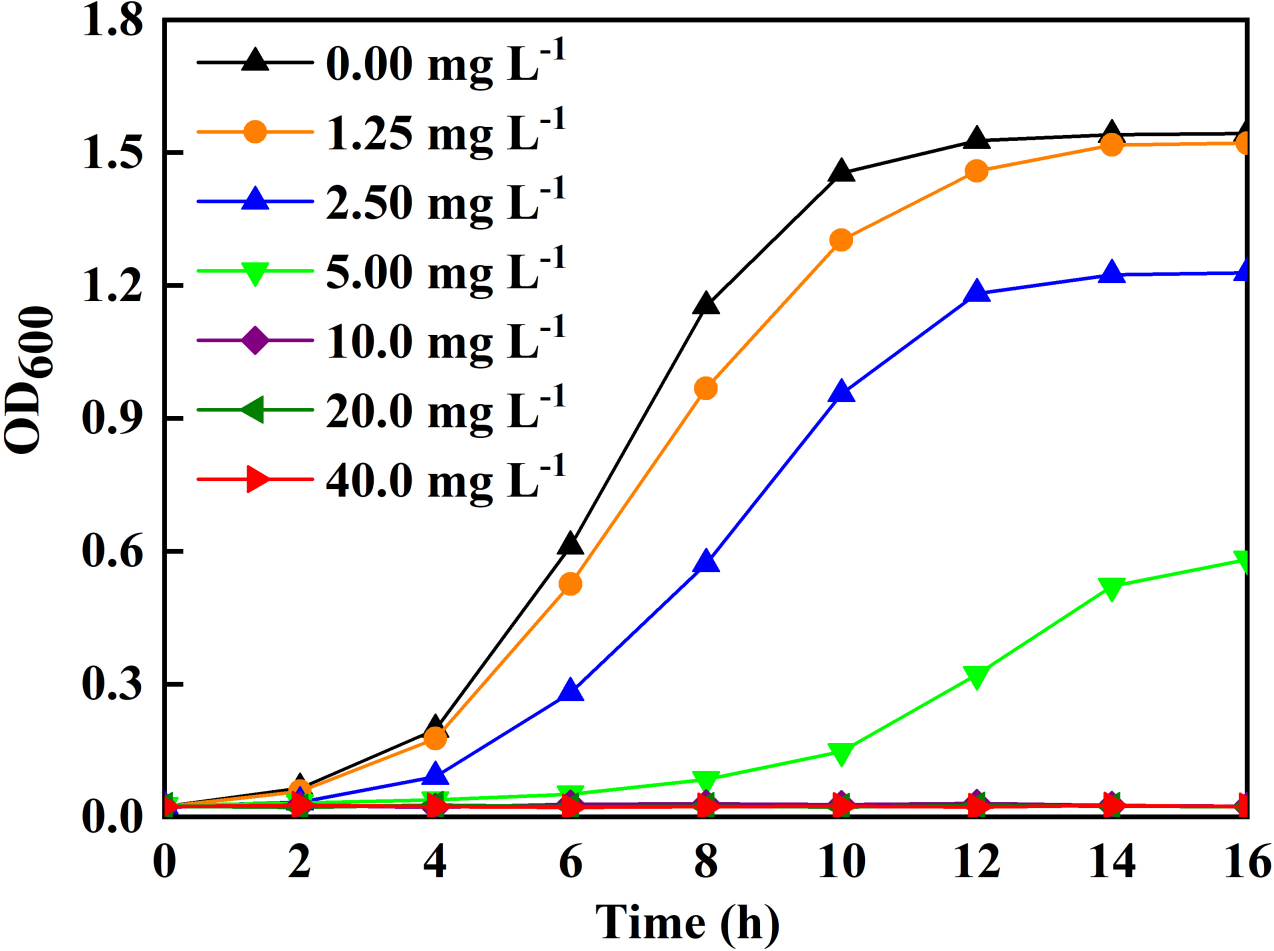
Ag-NPs on growth of the bacterial population isolated from the stem-ends of cut tree peony ‘Luoyang Hong’.

**Figure 5:**
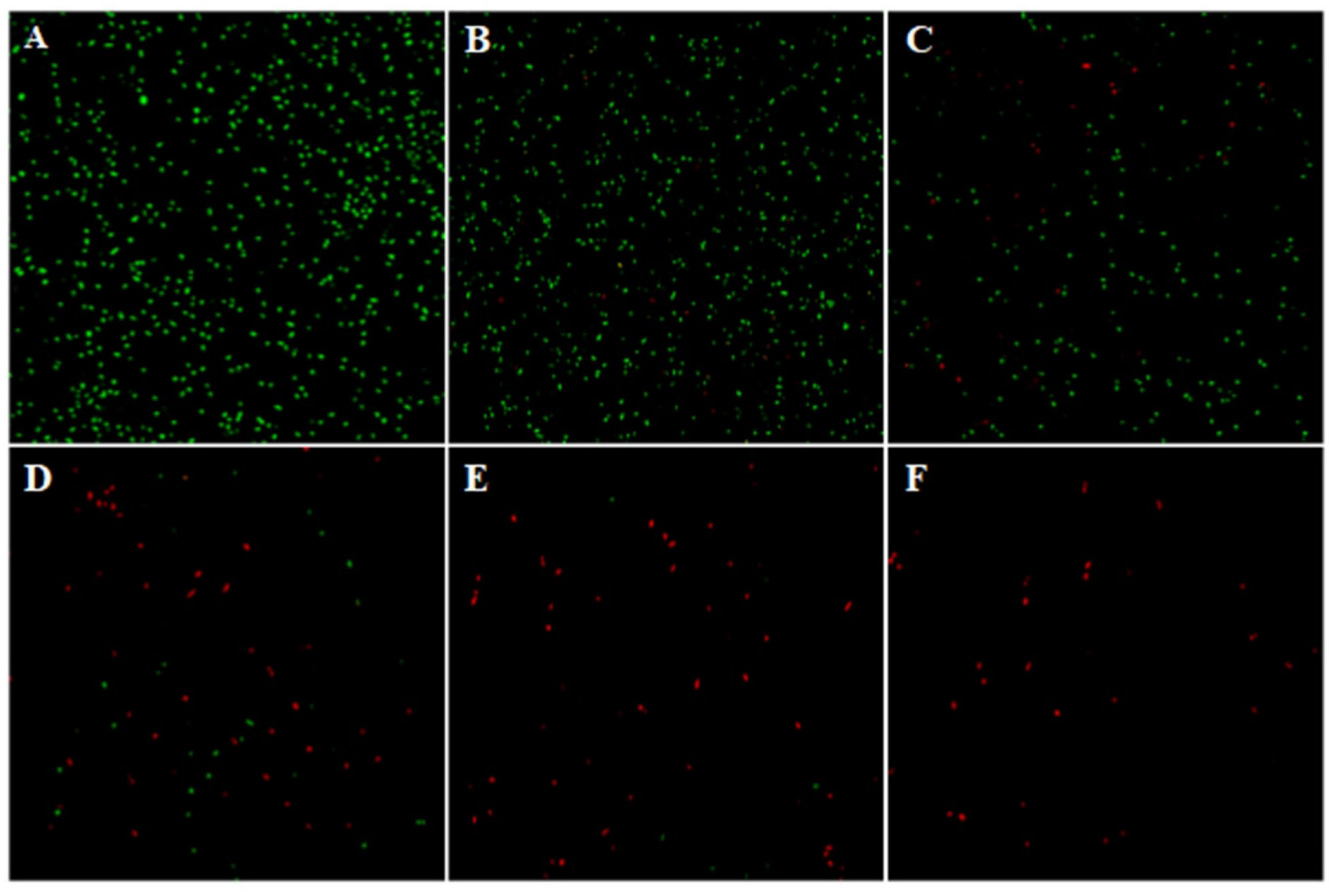
CLSM images for life (green)/dead (red) the bacterial population isolated from the stem ends of cut tree peony ‘Luoyang Hong’ in Ag-NPs co-culture for 24h. **(A)** 0 mg L^−1^, **(B)** 1.25 mg L^−1^, **(C)** 2.5 mg L^−1^, **(D)** 5 mg L^−1^, **(E)** 10 mg L^−1^, and **(F)** 20 mg L^−1^.

The Ag-NPs effects on bacterial growth were evaluated *in vitro* (**Figure 4**). The higher the Ag-NPs, the stronger the inhibitory effect. The culture broth had no turbidity when the concentration of Ag-NPs reached 10 mg L^−1^, indicating 10 mg L^−1^ as the MIC. To further confirm the inhibitory efficacy of Ag-NPs, the CLSM assay was conducted using SYTO9 (stained life cells and dead cells) and PI (stained dead cells) as fluorescent nucleic acid dyes. The results revealed the distribution of dead/viable bacteria under the different concentrations of Ag-NPs treatment for 24 h (**Figure 5**).

### 3.4 Pretreatment with Ag-NPs on vase performance

**Figure 6:**
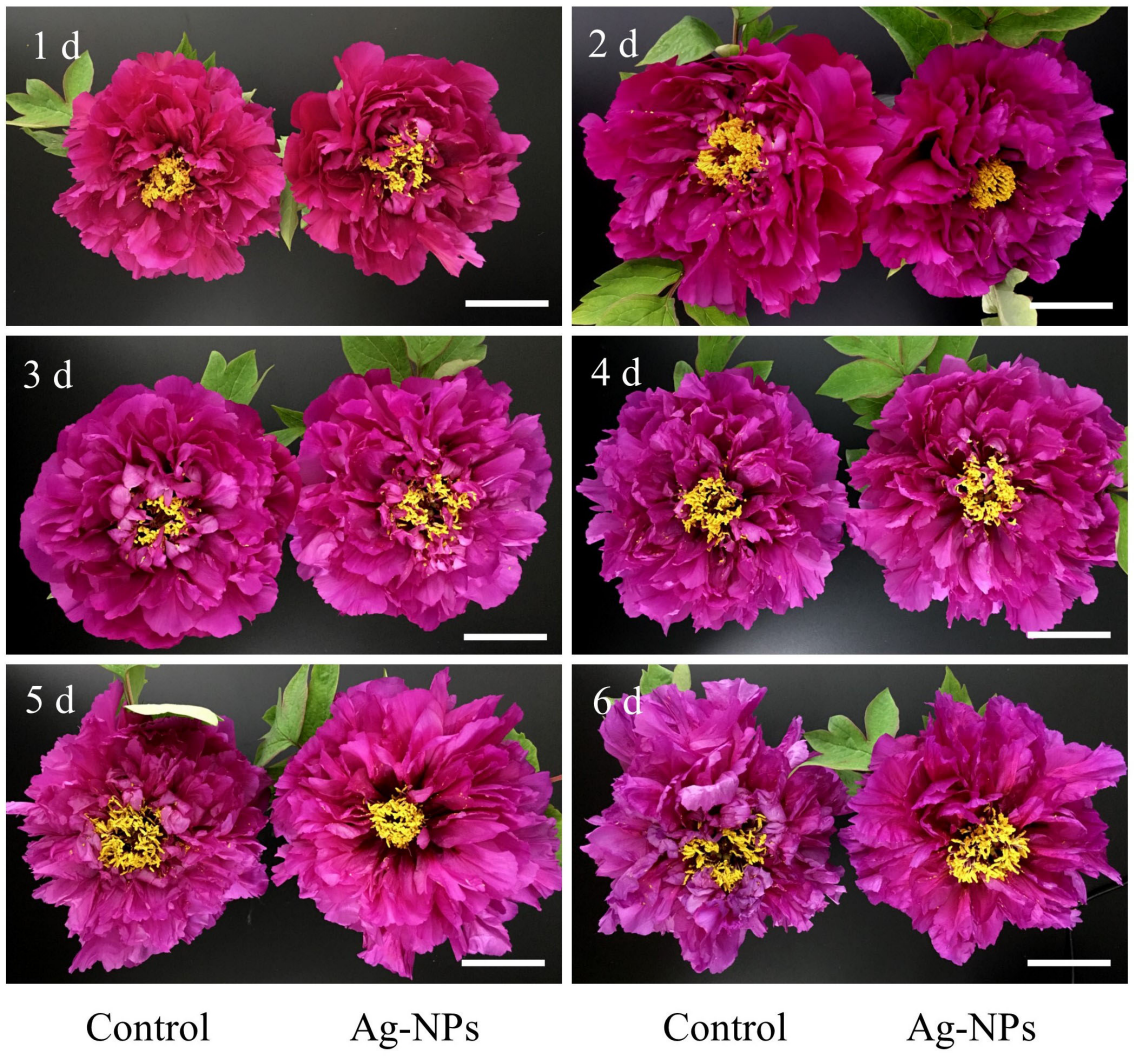
Pretreatment with 10 mg L^−1^ Ag-NPs on ornamental quality performance of cut tree peony ‘Luoyang Hong’ flowers. Scale bars = 4 cm.

Pretreatment with 10 mg L^−1^ Ag-NPs aqueous solution improved vase the performance of the cut tree peony ‘Luoyang Hong’ flowers (**Figure 6**). The results showed that the pretreatment and the control entered the optimum viewing period on day 1 (Stage IV). On day 4, the control petals were wilted, whereas those of the pretreated cut flowers were still in the optimum viewing period. Nearly all the pretreated cut flowers retained good ornamental quality on day 5.

**Figure 7:**
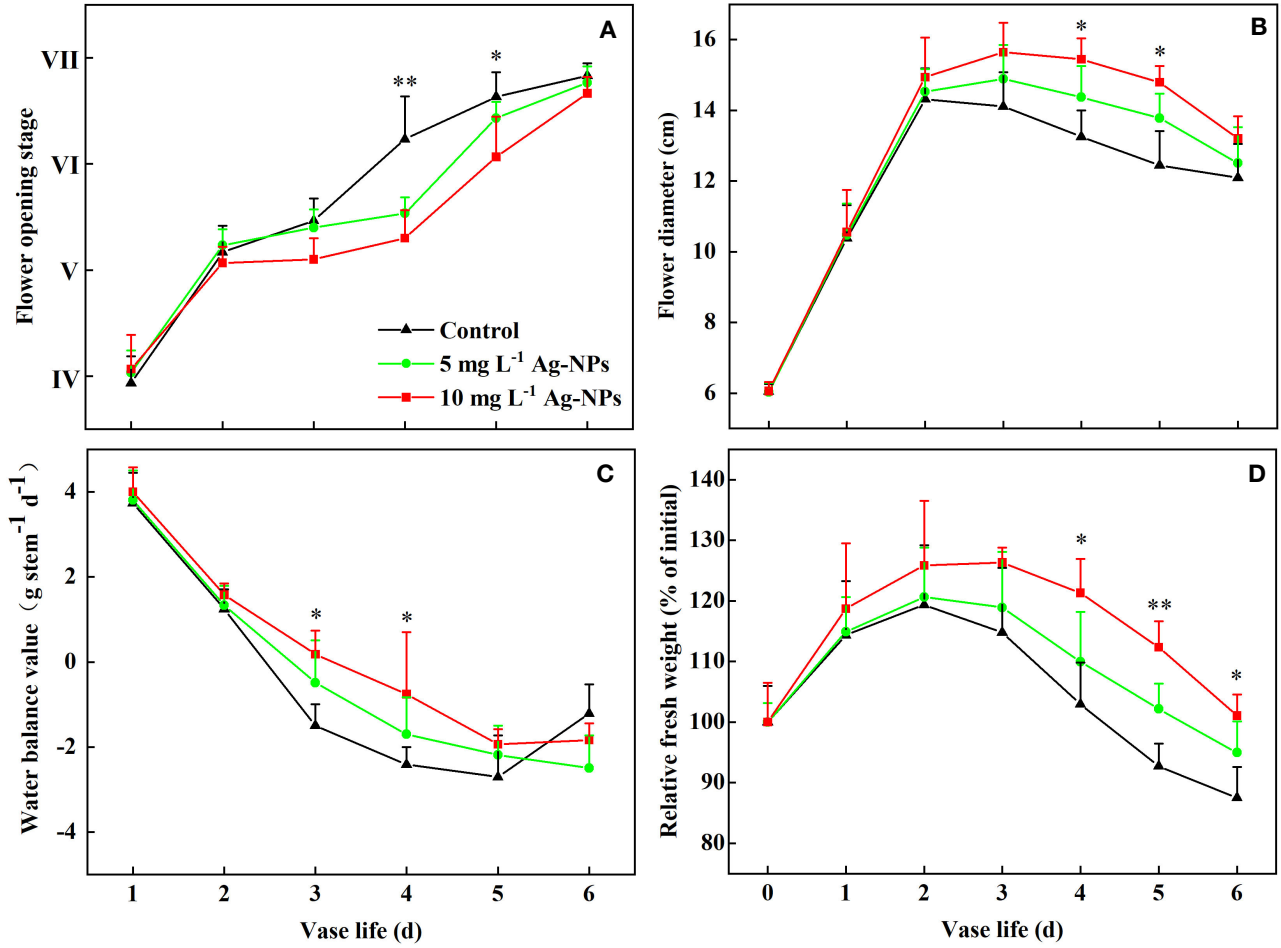
Flower opening stage **(A)**, flower diameter **(B)**, water balance **(C)**, and RFW **(D)** of cut tree peony ‘Luoyang Hong’ flowers pretreated for 24 h with DW (Control) or 5 or 10 mg L^−1^ of Ag-NPs (n =10). The vertical bars indicate LSD_0.05_ for the treatment comparisons. * shows significance between 10 mg L^−1^ Ag-NPs pretreated and control (*p* ≤ 0.05). ** shows significance between both of Ag-NPs pretreated and control (*p* ≤ 0.05).

Pretreatments with Ag-NPs aqueous solution extended the vase life of the cut tree peony ‘Luoyang Hong’ flowers (**Figure 7A**). The pretreated samples with 5 and 10 mg L^−1^ Ag-NPs aqueous solution reached stage VI at 4.6 and 5.1 d, respectively. The optimum viewing periods were 3.6 and 4.1 d, respectively, which was 0.9 and 1.4 d longer than the control on average.

The diameter of the flowers pretreated with Ag-NPs aqueous solution increased during the early stage of vase life and reached the maximum value on day 3, Still, the control was on day 2 (**Figure 7B**). The diameter of the flowers pretreated with 10 mg L^−1^ Ag-NPs aqueous solution had the highest value (15.7 cm) and was 9.3% larger than the control. Pretreatment with Ag-NPs aqueous solution under both concentrations also maintained the floral diameter at the later stage (4−6 d) compared with the control.

During the vase life period (1−5 d), the water balance declined for the control and the pretreatment with Ag-NPs aqueous solution. However, the rate of decline for the pretreatment with Ag-NPs aqueous solution was slower (**Figure 7C**). The pretreated cut flowers with 5 and 10 mg L^−1^ Ag-NPs aqueous solution reached a water balance value of 0, about 0.3 d and 0.6 d later than the control, respectively. On day 6, the water balance for the control increased rapidly, while the pretreated cut flowers maintained the water balance.

The RFW of the cut tree peony ‘Luoyang Hong’ flowers changed similarly for the control and the pretreatment with Ag-NPs aqueous solution (**Figure 7D**). The RFW reached the maximum value on day 2 for the control and the pretreatment with 5 mg L^−1^ Ag-NPs aqueous solution, but on day 3 for the pretreatment with 10 mg L^−1^ Ag-NPs aqueous solution. The RFW of the pretreatment with 10 mg L^−1^ Ag-NPs aqueous solution increased by 6.5% and 11.5% on days 2 and 3, respectively, compared with the control. The RFW of the pretreatment with Ag-NPs aqueous solution was much higher than that of the control in late vase life (4−6 d).

### 3.5 Pretreatment with Ag-NPs on physiological parameters

**Figure 8:**
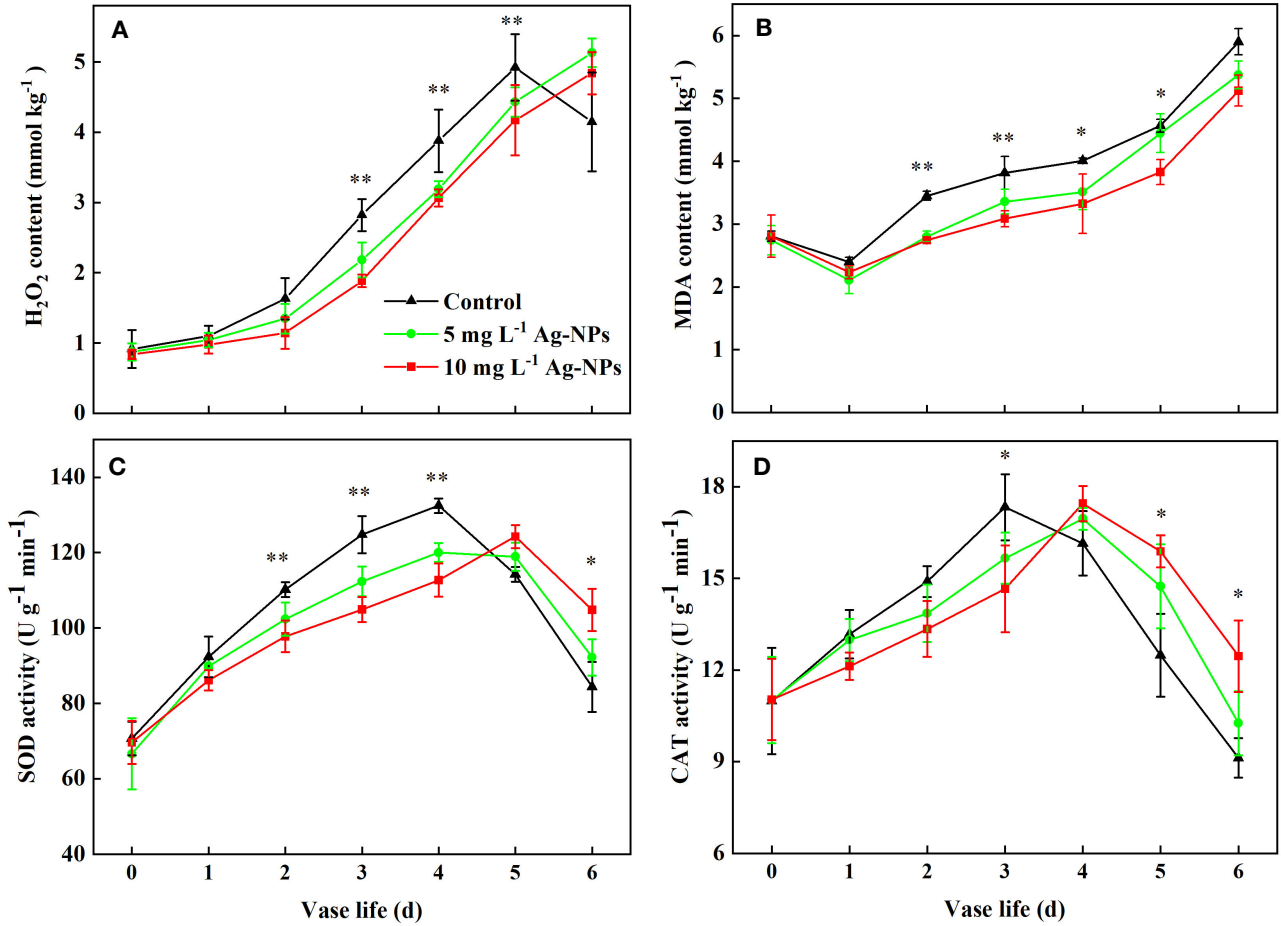
H_2_O_2_ contents **(A)**, MDA contents **(B)**, SOD activities **(C)**, and CAT activities **(D)** in petals tissue of cut tree peony ‘Luoyang Hong’ flowers during the vase period (n =5). Vertical bars indicate LSD_0.05_ for the treatment comparisons. * shows significance between 10 mg L^−1^ Ag-NPs pretreated and control (*p* ≤ 0.05). ** shows significance between both of Ag-NPs pretreated and control (*p* ≤ 0.05).

The H_2_O_2_ content for the control and the pretreatment with Ag-NPs aqueous solution changed little in the early stage (0−1 d) and rapidly increased from day 2 to day 5. The H_2_O_2_ level for the pretreatment with Ag-NPs aqueous solution was lower than the control at stage 2−5 d. The H_2_O_2_ level for the control decreased on day 6, which may have been caused by petal wilts (**Figure 8A**). For pretreatment with Ag-NPs aqueous solution, the H_2_O_2_ levels were still raised at that time.

The MDA levels in all petals were the lowest on day 1, then increased in the vase period (2−6 d). The MDA content for the pretreatment with Ag-NPs aqueous solution was lower than the control during the entire vase life (**Figure 8B**). In particular, the MDA content of petals for the pretreatment with 10 mg L^−1^ Ag-NPs aqueous solution was reduced by 20.5%, 19.2%, 17.0% and 16.1%, compared with the control at the vase period (2−5 d), respectively.

Pretreatment with Ag-NPs aqueous solution could affect antioxidant enzyme activity in petals (**Figures 8C, D**). The SOD activity in petals pretreated with Ag-NPs aqueous solution did not differ from the control in the first two days. At the stage of senescence (2−4 d), the SOD activities for the pretreatments with 5 and 10 mg L^−1^ Ag-NPs aqueous solution were 8.1% and 14.2% lower than the control. The levels of SOD activity for the pretreatments decreased at the late vase life (5−6 d), but were higher than the control. The CAT activity in the control petals reached the maximum value on day 3 of the vase life, and decreased gradually. Compared with the control, the CAT activity for the pretreatment with Ag-NPs aqueous solution was lower in the early vase life (0−3 d). Pretreatment with 10 mg L^−1^ Ag-NPs aqueous solution resulted in lower CAT activity on day 3 and by 15.4% compared to the control. However, at the late stage (4−6 d), the CAT activity for the pretreatment with Ag-NPs aqueous solution was higher than the control.

### 3.6 Ag^+^ distribution in stems

**Figure 9:**
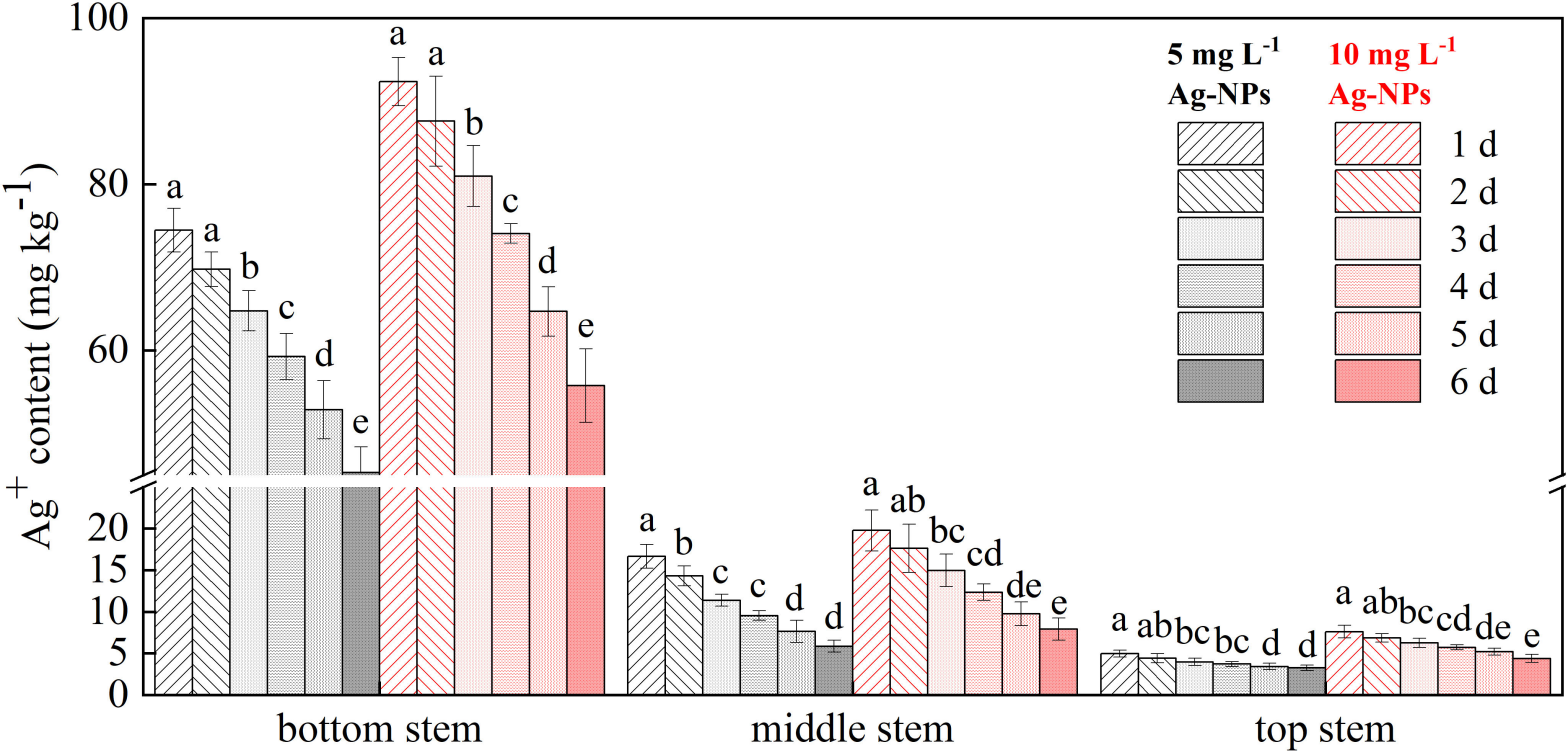
Concentrations of Ag in cut tree peony ‘Luoyang Hong’ bottom stem, middle stem and top stems during the vase period (n = 5). The vertical bars indicate the LSD_0.05_ for treatment comparisons. Different small letters meant significant difference among vase life (d) at 5 mg L^−1^ Ag-NPs and 10 mg L^−1^ Ag-NPs, respectively.

The distribution of Ag^+^ in different parts of the cut ‘Luoyang Hong’ stem varied for pretreatment with 5 and 10 mg L^−1^ Ag-NPs aqueous solution, in contrast, Ag^+^ had not been detected in control stems because of its low content (**Figure 9**). The bottom stem had the highest amount of Ag^+^, and the lowest content of Ag^+^ was found in the top stem. On the day 3, the Ag^+^ content of bottom stems significantly decreased than the previous two days. Additionally, the Ag^+^ content of stems pretreated with 10 mg L^−1^ Ag-NPs aqueous solution was higher than that of cut tree peony ‘Luoyang Hong’ pretreated with 5 mg L^−1^ Ag-NPs aqueous solution in the vase period, and their content decreased as the vase period went on.

### 3.7 Pretreatment with Ag-NPs on bacterial growth in xylem vessels

**Figure 10:**
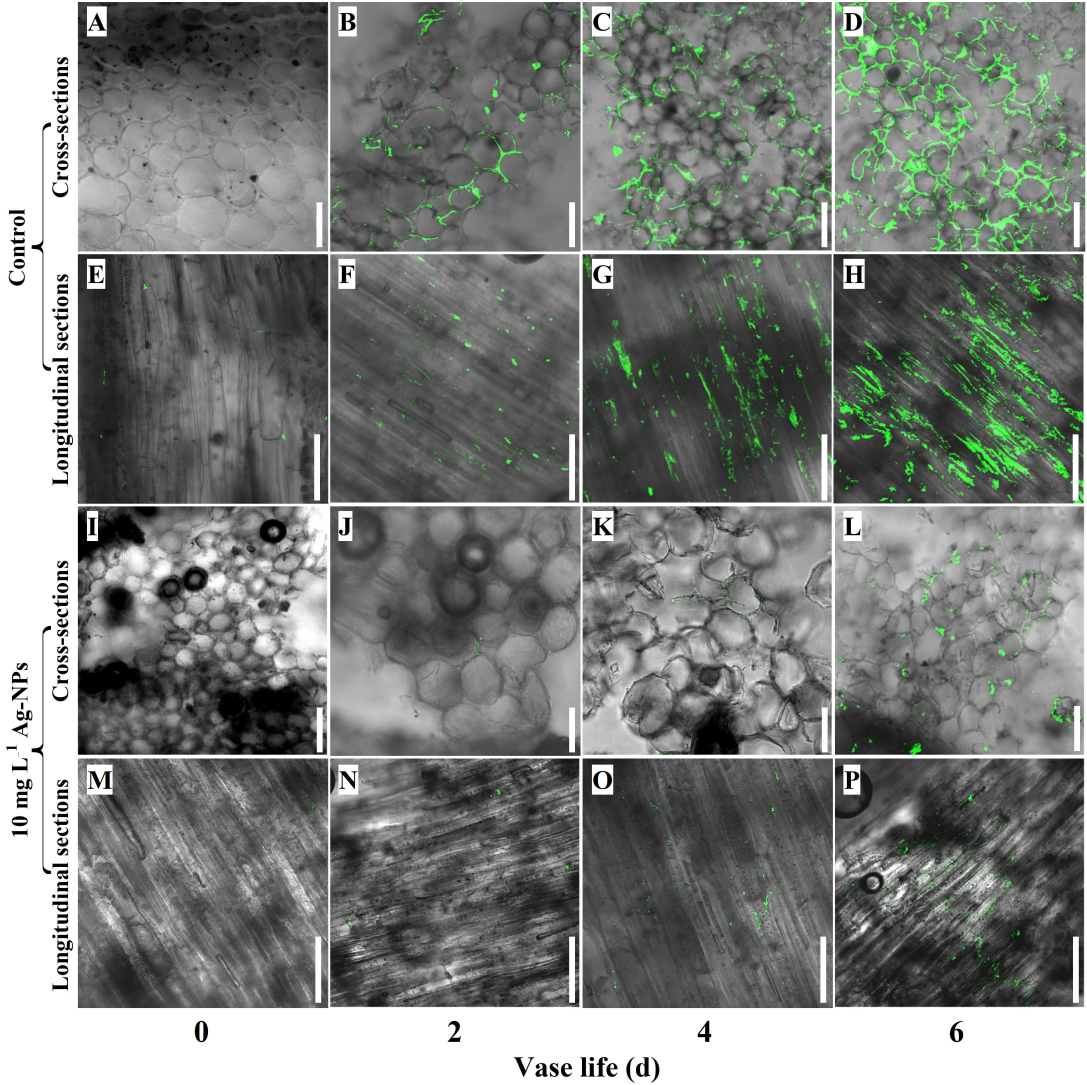
CLSM observations of bacterial reproduction in xylem blockage of cut tree peony ‘Luoyang Hong’ stems. **(A–H)** The control treatments were directly placed in deionized water and observed on days 0, 2, 4 and 6, respectively, of the vase life. **(I–P)** The treatments with Ag-NPs aqueous solution for 24 h before being placed into deionized water and observed on days 0, 2, 4 and 6, respectively, of the vase life. Scale bars = 50 µm.

CLSM observations showed that pretreatment with 10 mg L^−1^ Ag-NPs aqueous solution reduced bacterial reproduction in the xylem vessels of cut tree peony ‘Luoyang Hong’ compared with the control (**Figure 10**). Only a few bacterial cells could be found in the longitudinal sections and cross-sections of cut stems on day 0 (**Figures 10A, E, I, M**). The bacterial cells could be evidently discovered on day 2 in control, (**Figures 10B, F**). The bacterial cells had covered the surfaces of longitudinal sections and cross-sections on day 4 and day 6 (**Figures 10C, D, G, H**). In contrast, on day 2 and day 4, a small number of bacterial cells were observed in the longitudinal sections and cross-sections of cut stems pretreated with Ag-NPs aqueous solution (**Figures 10J, K, N, O**), even on day 6 (**Figures 10L, P**).

**Figure 11:**
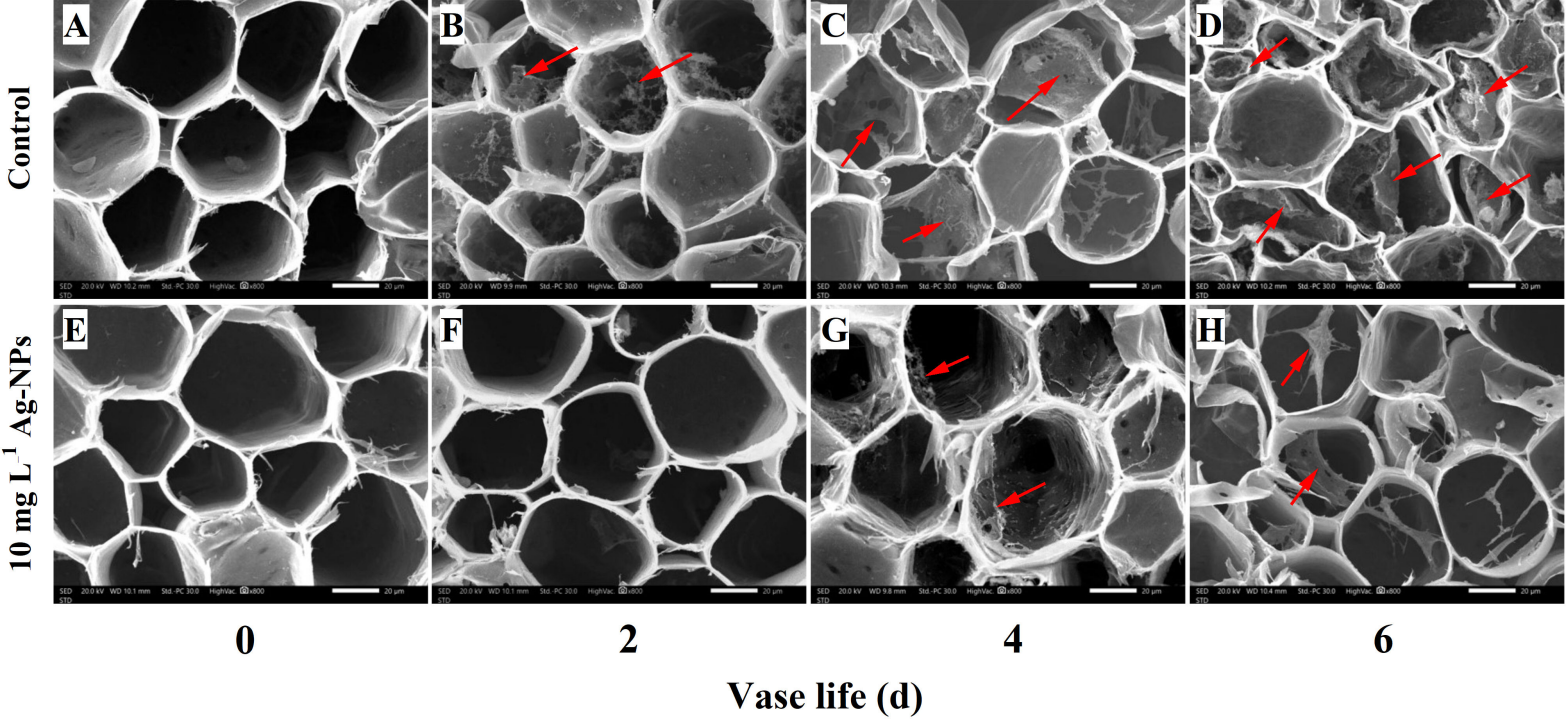
SEM images of bacterial proliferation on the stem ends of cut tree peony ‘Luoyang Hong’ flowers during the vase period. Red arrows indicate the bacteria and the biofilm. **(A–D)** The control treatments were directly placed in deionized water and observed on days 0, 2, 4 and 6, respectively, of the vase life. **(E–H)** The treatments with Ag-NPs aqueous solution for 24 h before being placed into deionized water and observed on days 0, 2, 4 and 6, respectively, of the vase life. Scale bars = 20 µm.

The SEM images showed that no visual bacteria appeared on the cross-sections and in xylem vessels of control (**Figure 11A**) and pretreated stem ends on day 0 (**Figure 11E**). On day 2, bacteria were evident on the stem ends of the control (**Figure 11B**) and few bacteria were observed on the pretreated stem ends (**Figure 11F**). On day 4 and day 6, there were mainly covered with bacteria and the bacterial proliferation accelerated resulting in biofilm under control conditions (**Figures 11C, D**). In contrast, a small number of bacterial cells were observed on the cross-sections and in xylem vessels of cut stems pretreated with Ag-NPs aqueous solution on day 4 and day 6 (**Figures 11G, H**).

## 4 Discussion

Cut tree peony ‘Luoyang Hong’ have a shorter vase life than rose, carnation, and lily (Hassan et al., 2014; Basiri et al., 2022). Adding biocides to the vase solution is an effective way to extend the vase life. A vase solution containing 50 mg L^−1^ ClO_2_ increased the vase life of cut ‘Luoyang Hong’ by 2.12 d (Nian et al., 2017). Pretreatment with 0.01 μmol L^−1^ rapamycin for 2 h could also prolong the vase life of cut ‘Luoyang Hong’ from 4.8 to 5.6 d (Wang et al., 2020c). Nanotechnology has the potential to enhance the vase life of cut flowers by using biosynthetic nano-antibacterial agents. In the literature, the vase life of cut *D. caryophyllus* was prolonged from 5.8 to 8.9 d by using biosynthetic Ag-NPs at 250 mg L^−1^ (Xia et al., 2017). In China, *E. ulmoides* is a widely used medicinal plant (Lü et al., 2017). It is rich in bioactive components capable of scavenging free radicals and performing antibacterial functions (Zhu and Sun, 2018; Wang et al., 2020b). Green synthesized Ag-NPs using the bark extract of *E. ulmoides* have shown efficient inhibitory activity against *Escherichia coli* and *Staphylococcus aureus* (Lü et al., 2017). Our study presented a simple and efficient procedure for the green synthesis of Ag-NPs using the leaf extract of *E. ulmoides*. The green synthesized Ag-NPs showed potent inhibitory activity against bacterial populations isolated from cut ‘Luoyang Hong’ stem *in vitro* (**Figures 4**, **5**). Several studies have shown that biosynthesized Ag-NPs have an excellent ability to suppress bacterial reproduction in the vase solution and at the stem ends of cut flowers (Carrillo-López et al., 2016; Pang et al., 2022). It was also reported that biosynthetic Ag-NPs using *A. annua* L. callus extracts could reduce bacterial blockage at the stem ends of *A. annua*, thus extending the vase life (Xia et al., 2017). Our study also found that the pretreatment with green synthesized Ag-NPs inhibited bacterial growth in cut tree peony ‘Luoyang Hong’ xylem vessels, resulting in longer vase life, optimum viewing period and excellent ornamental value.

Flower diameter increased initially and decreased later during senescence and postharvest development in cut tree peony ‘Luoyang Hong’ (Shi et al., 2015). Our research showed that the flower diameter pretreated with Ag-NPs aqueous solution was always larger than that of the control during the vase period (**Figure 7B**), which was consistent with the reports in *P. lactiflora* Pall. treated with 30 mg L^−1^ Ag-NPs (Zhao et al., 2018). Petal growth is mainly based on cell expansion, which requires water to enter the cell. Cut flowers are affected directly by a disruption of water uptake (Chen et al., 2013). Therefore, solving the xylem occlusion caused by bacteria could improve water uptake and increase flower diameter (Liu et al., 2009; Liu et al., 2012; Naing et al., 2017). The water balance of cut flowers is a critical indicator of vase life, ornamental quality, and freshness after harvest. Some correlation existed between water uptake and bacterial growth in cut flower stem ends, according to Xia et al. (2017). In cut carnation ‘Omea.’, Ag-NPs increased vase life and reduced water loss because of the antibacterial properties, which would inhibit bacterial reproduction in xylem vessels (Naing et al., 2017). Our study showed the same trends in the water balance and RFW for the pretreatment with Ag-NPs aqueous solution. The cut tree peony ‘Luoyang Hong’ pretreated with Ag-NPs aqueous solution also had higher RFW and better water balance than the control (**Figures 7C, D**). The results indicated that the pretreatment with Ag-NPs improved water transportation and increased FRW by inhibiting bacterial reproduction in xylem vessels.

Senescence of cut flowers is affected by an increase in membrane lipid peroxidation and free radicals, including H_2_O_2_, as well as its final product MDA in petals, such as rose, carnation, tree peony, and *Strelitzia reginae* L (Hassan et al., 2014; Shi et al., 2015; Aalifar et al., 2020; Thakur et al., 2022). Antioxidant defense systems such as SOD, CAT, and POD are employed in plant cells that can be protected from the danger of reactive oxygen species (ROS) (Shi et al., 2015; Hasanuzzaman et al., 2020; Zulfiqar and Ashraf, 2021; Seyed Hajizadeh et al., 2023). Many studies have shown that Ag-NPs play an important role in limiting H_2_O_2_ levels and reducing MDA production by inhibiting bacterial growth at the stem ends, thereby extending the life of cut flowers (Hassan et al., 2014; Rashidiani et al., 2020; Anvari et al., 2022). The H_2_O_2_ and MDA contents in pretreated petals were lower than that in control in our study (**Figures 8A, B**), which was in line with previous reports (Zhao et al., 2018; Das and Mandal, 2020; Rabiza-Świder et al., 2020a; Rashidiani et al., 2020). The SOD and CAT activities for the control and pretreatment with Ag-NPs aqueous solution increased in our study early on and declined later on (**Figures 8C, D**). Compared with the control, the activities of protective enzymes in petals were lower during the previous four days and were higher during the last two days under the pretreatments with Ag-NPs aqueous solution. Researchers have observed a similar pattern in *Chrysanthemum morifolium* Ramat. cv. Maghi (Chakrabarty et al., 2007), *G. hortulanus* L. (Saeed et al., 2013), *P. lactiflora* Pall. (Zhao et al., 2018), and *S. reginae* L. (Thakur et al., 2022). There might be an explanation that the pretreatment with Ag-NPs aqueous solution produced lower tissue damage and slower senescence in cut tree peony ‘Luoyang Hong’ flowers.

During vase life, the biological macromolecules in the stem stimulate growth of endophytic bacteria, resulting in the blockage of stem ends and xylem vessels. Some researchers have demonstrated that nano antibacterial agents, such as silver, copper, and chitosan nanoparticles can inhibit bacterial growth and reduce stem blockage (Li et al., 2017; Spricigo et al., 2021). Pretreatment with Ag-NPs reduced bacterial growth in the stem ends and increased water uptake, and RFW in cut *G. hybridus* ‘Eerde’ spikes (Li et al., 2017). It also proved that a vase solution with Ag-NPs alleviated xylem vessel blockage by reducing bacterial growth and biofilm formation of cut *D. caryophyllus* L. in association with enhanced vase life and solution uptake (Lin et al., 2019). CLSM observations in the study confirmed that the xylem vessels of cut tree peony ‘Luoyang Hong’ were entirely covered by living bacteria on day 4 and day 6 in control (**Figures 10C, D, G, H**). Pretreatment with Ag-NPs reduced bacterial reproduction in the stem ends and xylem vessels (**Figures 10K, L, O, P**). Meanwhile, according to SEM observations, the Ag-NPs pretreatment significantly reduced bacterial proliferation and biofilm formation on the stem ends of cut ‘Luoyang Hong’ as compared to the control (**Figure 11**). The results demonstrated efficacy of pretreatment with Ag-NPs aqueous solution in alleviating bacteria-related blockage of the stem ends of cut tree peony ‘Luoyang Hong’, which probably improved the water uptake and RFW, resulting in longer vase life and optimum viewing period (Li et al., 2017; Liu et al., 2021; Thakur et al., 2022). In addition to inhibiting bacterial proliferation at stem ends, Ag-NPs also enter the xylem to inhibit bacterial growth and biofilm formation under Ag-NPs pretreatment (**Figure 11**). The Ag-NPs accumulated in tree peony ‘Luoyang Hong’ stems were detected, and Ag^+^ mostly remained within the bottom stem of Ag-NPs aqueous solution pretreated cut tree peony ‘Luoyang Hong’ flowers during the vase period (**Figure 9**). These results were the same as the results reported for cut *D. caryophyllus* flowers and cut *P. lactiflora* Pall treated with Ag-NPs (Koohkan et al., 2014; Zhao et al., 2018; Thakur et al., 2022). Interestingly, endophytic bacteria grew easily at the stem end, and the presence of Ag^+^ mainly remained within the stem bottom, indicating that Ag-NPs could exert their antibacterial effects continuously and alleviate blockage (Zhao et al., 2018).

## 5 Conclusion

A simple and efficient method for green synthesis of Ag-NPs using the leaf extract of *E. ulmoides* was developed. The Ag-NPs aqueous solution showed excellent antibacterial activity *in vitro* against bacterial populations isolated from cut tree peony ‘Luoyang Hong’ stem ends. Moreover, the bacterial growth in the xylem vessels of cut tree peony ‘Luoyang Hong’ was reduced by pretreatment with 10 mg L^−1^ Ag-NPs aqueous solution for 24 h. Consequently, pretreatments with Ag-NPs aqueous solution maintained the water balance and extended the vase life of cut tree peony ‘Luoyang Hong’ to improve postharvest quality and ornamental value. To our knowledge, it is the first time that the green synthesized Ag-NPs have been applied to cut tree peony. Therefore, the research could provide a novel, practical and environment-friendly method for preserving cut tree peony and other cut flowers.

## Data availability statement

The raw data supporting the conclusions of this article will be made available by the authors, without undue reservation.

## Author contributions

ZM and JL designed the experiments, wrote and revised the article. WG, WY, and JW carried out the experiments. KZ and WY analyzed the data and took photographs. All authors contributed to the article and approved the submitted version.
